# Phenomenology for primary care researchers

**DOI:** 10.4102/phcfm.v17i2.4946

**Published:** 2025-05-28

**Authors:** Robert Mash, Febisola Ajudua, Sebaka Malope, Doreen Kaura

**Affiliations:** 1 Division of Family Medicine and Primary Care, Faculty of Medicine and Health Sciences, Stellenbosch University, Cape Town, South Africa; 2 Department of Family Medicine and Primary Care, Faculty of Health Sciences, Nelson Mandela University, Gqeberha, South Africa; 3 Lesotho Boston Health Alliance, Leribe, Lesotho; 4 Ministry of Health, Maseru, Lesotho; 5 Department of Nursing and Midwifery, Faculty of Medicine and Health Sciences, Stellenbosch University, Cape Town, South Africa

**Keywords:** primary care, methodology, phenomenology, descriptive phenomenology, interpretive phenomenology, research methods

## Abstract

Primary care researchers often turn to qualitative methodologies to explore people’s perspectives and experiences. Phenomenology is appropriate when the focus is on lived experiences, rather than ideas, beliefs, opinions or perceptions. Phenomenology has its roots in German philosophy and the social sciences, and doctoral students as well as researchers in the health sciences may struggle to understand the paradigm and apply it practically. This article attempts to make sense of the paradigm and two of its key threads, namely descriptive and interpretive phenomenology. The key principles of both approaches and the practical methodological steps are outlined. In addition, examples are given, and the two approaches are compared. Finally, the article discusses trustworthiness and quality criteria in phenomenology.

## Introduction

Primary care researchers often turn to qualitative methodologies to explore questions related to people’s ideas, beliefs, opinions, perceptions, satisfaction and experiences. Phenomenology is one of the qualitative methods that such researchers are embracing, and yet it is frequently misunderstood. It is not uncommon for peer reviewers to question whether the study design is really phenomenology. In this article, we outline the phenomenological approach to qualitative research for primary care and family medicine researchers embedded in the health sciences. We have attempted to plot a practical path that doctoral researchers can follow while maintaining connection to the theoretical and philosophical roots.

A phenomenon has been defined ‘as something that exists and can be seen, felt, tasted, etc., especially something unusual or interesting’.^1^ For primary care researchers, this might be how patients experience a condition or health care, for example, the experience of living with diabetes or attending the clinic when you are pregnant. It could also focus on the experience of healthcare workers, for example, the experience of working in a field hospital during the coronavirus disease 2019 (COVID-19) pandemic. Understanding subjective experiences can contribute to improved holistic care.

Research questions might be phrased as ‘What is it like to experience …?’ or ‘How do people experience …?’ and then focus on the phenomenon of interest.

## Phenomenology as a research paradigm

Phenomenology was conceptualised by German philosophers in the 19th century who were interested in exploring people’s lived experiences.^2^ They were moving away from the positivist paradigm, which remains the dominant paradigm in health sciences, towards what is now called the interpretive-hermeneutic paradigm. Much of the methodological discourse on phenomenology emanates from the social sciences and has only more recently been embraced by the health sciences, particularly in nursing.

In this paradigm, reality is not seen as a single objective truth that can be measured but as multiple subjective truths that are socially constructed and can be observed, described, explored and interpreted.^3^ The same phenomenon can be experienced differently by people, and all these experiences are valid. New knowledge is constructed through interacting with these multiple realities and interpreting them. The methodology, therefore, is characterised by an approach that is explorative, creative and reflective and seeks to understand what people experience regarding the phenomenon of interest.

It should be emphasised that the focus is on the nature of this lived experience and what it means to people, and not on the causes of the experience or the extent of the phenomenon. For example, for people living with diabetes, the focus would be on people’s experience of the condition and not on why they have diabetes or how many people have diabetes. Likewise, if the focus is on people’s opinions, beliefs or perceptions, this would not be phenomenology. For example, asking healthcare workers their perceptions of why people struggle to use insulin would not be phenomenology, as they are not reporting on their own lived experience. Such study designs are often labelled as exploratory descriptive qualitative studies. Primary care and family medicine researchers, however, tend to be practical and are usually not interested in a phenomenon for its own sake but because the findings will have implications for improving people’s health and wellness, the quality of care or patient safety.

Within phenomenology, there are also different traditions and approaches. Descriptive (also known as transcendental) and interpretive (also known as hermeneutic) phenomenology will be described in this article.^4^ One of the key differences in these traditions is that descriptive phenomenology intends to describe the essential characteristics or essence of a particular phenomenon. There is a belief that the essential essence can be distilled and described. On the other hand, interpretive phenomenology intends to explore the meaning of the phenomenon to people in a particular historical and social context while accepting that such meaning is dynamic and forever changing. Table 1 juxtaposes key methodological differences between these approaches, which are further described in the following sections.

**TABLE 1:** Comparing descriptive and interpretive methodologies.

Key aspects of methodology	Descriptive	Interpretive
Aim and objectives	Understand and describe a phenomenon by exploring the lived experience of people	Explore how people make sense of a phenomenon by exploring the lived experience of people
Understanding of truth	The universal characteristics or essences of a phenomenon can be described	How people make sense of a phenomenon can be interpreted in a particular historical and social context that is always changing
Understanding of consciousness	Our consciousness is always intentionally focused on an object, event or situation in the world	Our consciousness and objects, events or situations in the world are inseparable
Relationship of the researcher to the research process	The researcher removes or ‘brackets’ their experiences, assumptions and presuppositions from the research process	The researcher acknowledges that they are inseparable from the research process and exploration of the phenomenon
Reflexivity	The researcher must describe how they bracketed off their own experiences, assumptions and presuppositions before exploring the phenomenon	The researcher should describe how they continuously reflected on their own experiences, assumptions and presuppositions as they explored the phenomenon
Study population, sample size and sampling	Purposeful sampling of individuals rich in the lived experience of the phenomenon with sufficient saturation of data	Purposeful sampling of individuals rich in the lived experience of the phenomenon with sufficient saturation of data
Data collection	Semi-structured or unstructured interviews	Unstructured interviews may be preferred
Data analysis	Thematic analysis following, for example, Colaizzi or Giorgi	Thematic analysis following the steps and hermeneutic circle described, for example, Pietkiewicz and Smith

## Descriptive phenomenology

Descriptive phenomenology intends to explore people’s conscious experience of a phenomenon and describe its critical or universal components. A key principle is called reduction, where the researcher attempts to remove all assumptions, judgements and presuppositions about the phenomenon and to focus on describing the direct, immediate, lived experience and get to the universal essence of the phenomenon. This is also referred to as bracketing or removing one’s own perspective from the research process.

Therefore, in data collection and analysis, the researcher tries to ‘bracket’ or remove their own experiences, biases or assumptions from the process (also referred to as epoché in some articles). They attempt to suspend their common-sense assumptions, predispositions based on past events or suppositions based on prior theories. When using descriptive phenomenology, begin by bracketing to be aware of and set aside your own assumptions. How such reflexivity was attempted should be described in the report. One way of doing this is to consciously adopt the stance of a stranger who has never encountered this phenomenon before when collecting data.

Intentionality is a key concept in descriptive phenomenology. Researchers understand that our consciousness does not exist in isolation, but our thoughts, perceptions and experiences are always directed towards an object, event or situation. Consciousness is always intentional and focused on something. The subject of the research with their consciousness is always connected to the object being experienced. The focus is on understanding how things appear to us and how they are experienced.

Both Giorgi and Colaizzi have described very similar processes in the analysis of data:^5,6^

- Step 1: Immerse yourself in the data as a whole by reading all the transcripts in their entirety.
- Step 2: Go back to each transcript and extract significant statements or identify meaning units that reflect different aspects of the participant’s experiences, thoughts, feelings and perceptions regarding the phenomenon.
- Step 3: Re-formulate these statements or transform these meaning units into a more concise and abstract summary of what was experienced.
- Step 4: Cluster these statements into groups or categories based on how they connect and reflect a broader pattern in the data.
- Step 5: Based on these categories, the researcher describes the key themes and identifies the essence of the phenomenon. The themes should be described comprehensively with a rich description.

Colaizzi goes on to recommend participant validation as the next step with a final integration of the themes into a coherent theoretical understanding of the phenomenon. Box 1 provides an example of a published study using descriptive phenomenology.

BOX 1 Example of a descriptive phenomenology study: lived experiences of Palestinian patients with COVID-19.^7^

**Table:** 

**Research question** How did Palestinian patients experience isolation for COVID-19 infection? The intention was to use the findings to improve healthcare policy and infrastructure in future pandemics.
**Study design** Descriptive phenomenology. The researchers wanted to understand and describe the essential characteristics of this experience.
**Setting** The setting was not fully described, and this may be a weakness of the published study. The participants were well described and came from the West Bank of Palestine.
**Study population, sample size and sampling** Academic nurses purposefully selected eligible participants who had been isolated in either healthcare centres or homes. The researchers planned to have 800 min of interview time to achieve saturation of themes (20 people × 40 min each). The only criteria listed were being an adult, being in isolation for 2–4 weeks and having a positive test for COVID-19.
**Data collection** Four academic nurse researchers from Palestine conducted semi-structured interviews via videotelephony software with the participants in their homes. An interview guide was developed, and participants were asked to recount in detail their experience from diagnosis to recovery. Researchers stated that they attempted to bracket their personal knowledge, experience and expectations during the research process.
**Data analysis** The researchers used Colaizzi’s approach to analysis with seven steps: Familiarisation: Each researcher read the transcripts several times to immerse themselves in the whole data. Identification of significant statements: Researchers identified all statements that shed light on different aspects of the experience (meaning units). Formulation of the meanings: The researchers re-formulated key aspects of the phenomenon from these statements (transformed meaning units). Clustering themes: The transformed meaning units were clustered and re-organised into groups or themes. Development of an exhaustive description: An exhaustive description of the phenomenon was created based on the themes. Production of the fundamental structure: Short, dense statements were created to describe the essential aspects of the phenomenon. Verification of the fundamental structure: Respondents were asked to validate these fundamental statements.
**Findings** Five key themes (and 16 subthemes) were presented: Emotions after learning about the infection Experiencing social exclusion and stigma The experienced symptoms Supportive treatments, herbs, rituals and social support Life after recovery

In some ways, this approach is a bridge between a more positivist mindset and an interpretivist one. The idea that a phenomenon has universal components and the attempt to separate oneself from it implies a belief that one can stand back and describe a phenomenon in isolation from oneself, the context and change over time. Interpretive phenomenology grew out of descriptive phenomenology and may have more utility for primary care researchers.

## Interpretive phenomenology

Interpretive phenomenology goes beyond describing the essence of the phenomenon to making sense of what it means for the people involved.^8^ Attention is also given to the historical, cultural and social context which influences how people attribute meaning. The importance of language is acknowledged, as meaning can literally be lost in translation.

In interpretive phenomenology, the idea that one can somehow separate oneself from the phenomenon and describe its essence from a distance is negated. The phenomenon is seen as dynamic, constantly evolving while existing in individual consciousness. One may be able to interpret meaning at a point in time, but one never arrives at a final truth. You are always on a journey, and the truth is a moving target interpreted from multiple realities.

For the same reasons, the intersubjectivity of the researcher and the researched is embraced, and bracketing is not seen as possible. While the researcher focuses on collecting and interpreting the data, they understand that they cannot isolate themselves from their own presuppositions and experiences. The researcher co-constructs their interpretation with participants while constantly being aware of and reflecting on their own experience and position. A reflective journal of one’s own history and experience with the phenomenon can be helpful. Sometimes one’s own position can be revealed by having a colleague interview you about your own experience or perceptions. In this approach, a researcher with prior experience in a phenomenon will be more suited to use interpretive phenomenology because they have insight into the phenomenon.

### Study setting

All research studies should describe the setting. In qualitative studies, this is particularly important to inform the reader and enable a judgement about the transferability of the findings. In interpretive phenomenology, the historical, cultural and social context in which people experience the phenomenon is of special importance.

### Study population, sample size and sampling

The study population should be defined in terms of who has a lived experience of the phenomenon. People are purposefully selected because they have a lived, unique and diverse experience of the phenomenon. The intention is maximum variation in the sampling to explore all the different ways in which people have experienced the phenomenon. The concept of saturation is used to determine the sample size so that people’s lived experiences are explored until no new insights are obtained.

### Data collection

Data is usually collected by interviewing people with the experience you are interested in. Unstructured in-depth interviews may be of more value than semi-structured ones, as there are no prior assumptions about the critical components of the experience that should be explored. The interview guide in semi-structured interviews will plan the topics to explore. In unstructured interviews, after the initial question, the interviewer will explore whatever aspects of the experience the person sees as important. Remember that the focus is on the actual experience, an account of how they lived through the experience, rather than their abstract conceptualisations about it. Ask for stories, accounts or specific examples of experiences. An opening question should be constructed that is aligned with this intention and stated in the methods when you report on the study. The goal is to allow participants to tell a story while expressing their thoughts, emotions and reflections and to provide deep insights into the phenomenon under study.

The interviewer must have good communication skills to enable an in-depth conversation while following the person’s story. Typically, an interview will last an hour. The communication style will be one of openness, acceptance, non-judgement and curiosity. Communication skills in active listening, open questions and clarification will be important. The interview will be recorded and a verbatim transcript will be created and checked.

### Data analysis

Data collection and analysis may be concurrent. In addition, follow-up interviews with the same people may validate their transcript, gather more data or check the interpretation. Researchers should be cautious of rushing to conclusions and should hold their initial analytical ideas lightly. Analysis will focus more on what is being experienced (events and emotions) and what it means to the individuals, rather than trying to uncover universal aspects of the phenomenon as in descriptive phenomenology. From an ethical perspective, the exploration of what the experience means for the person can raise difficult or challenging emotions, and psychological support may be needed or anticipated.^9^

All statements should be treated as equally important while developing meanings and clustering themes. No elements of the experience should be arbitrarily excluded because they seem bizarre, irrational or contradictory. The researcher should not edit or order the nature of people’s experiences to make them more coherent.

It is necessary to note that the choice of philosophical perspective, whether descriptive or interpretive, influences the choice of data analysis framework. In either case, qualitative data analysis software, such as ATLAS.ti, can assist the process and make the audit trail easy to follow. In broad terms, the data analysis process follows these steps^10,11^:

- Familiarise yourself with all the data. By listening again to the tapes as well as reading and re-reading the transcripts, you immerse yourself in the participants’ experiences.
- Return to each transcript individually. From the data, make notes on language, emotions, events and the emergent meanings of the experiences. Be aware of your own reactions and make notes to assist personal reflexivity.
- Identify initial emergent themes from your notes on the transcript that capture the core aspects of the experiences and what they mean.
- Search for connections between themes in the transcript and refine the themes through grouping, splitting, combining or discarding themes.
- Synthesise themes across all transcripts and ensure they are distinct and communicate the essence of the phenomenon and how people made sense of it.
- Write up the themes as a narrative that grounds them in the data and aligns with the research question and the principles of interpretive phenomenology (e.g. historical context, researcher’s reflexivity). The researchers must provide a rich, detailed account of participants’ experiences through direct quotations that illustrate the emergent meanings and themes. In the health sciences, the discussion section of an original research article is then used to discuss how these themes relate to existing literature.

Box 2 summarises a published example of interpretive phenomenology.

BOX 2 Example of an interpretive phenomenological study: Exploring women’s experiences with cultural practices during pregnancy and birth in Keiyo, Kenya.^12^

**Table:** 

**Research question** How do women experience cultural practices during pregnancy and birth in the Keiyo South constituency, Kenya? The intention was to develop more responsive healthcare and to optimise positive childbearing outcomes.
**Study design** Interpretive phenomenology. The researchers wanted to understand the meaning that women attached to the cultural issues that arose during their experience of pregnancy and birth.
**Setting** The researcher described in some detail the community setting, ethnic groups and different languages, as well as traditional and allopathic health services.
**Study population, sample size and sampling** Ten villages were purposively selected, and maximal variation purposeful sampling was used to select women in the third trimester to 3 months post-partum. The sample size was determined by saturation of themes, which occurred after 16 interviews, although 18 interviews were conducted in total.
**Data collection** The researcher was a midwife who grew up in the Keiyo-speaking community. She used her knowledge and insights of cultural practices to better explore and understand the women’s experiences. She did not attempt to bracket herself but reflected on her own background and presuppositions. She was fluent in the local languages.
Semi-structured individual interviews were conducted in the women’s homes or community settings using an interview guide. The opening question was ‘Tell me about your experiences with cultural practices during pregnancy and birth’. Interviews lasted up to 2 h, were audio-recorded, transcribed verbatim and checked for accuracy.
**Data analysis** Data collection and analysis were concurrent. The first six interviews were analysed (steps 1–3 below) and then analysis was repeated after each group of three additional interviews. The analysis could inform the questions and exploration of topics in subsequent interviews. ATLAS.ti software was used to assist with the analysis.
**Six steps of thematic analysis were used:** Familiarisation with the data by reading and re-reading the transcripts. Identification of codes and how they could be categorised. Coding of transcripts and initial potential themes identified. Steps 1–3 were repeated with each new group of transcripts. Final themes identified from the whole dataset. Themes were presented in a written report with illustrative quotes.
**Findings** Three key themes and several subthemes were presented: Encounters with sexual and reproductive health: abortion practices, female genital mutilation practices and sexual intercourse practices Pregnancy and birth encounters: child-bearing-related advice, companionship during pregnancy and birth What women want: companionship, effective communication and collaborative care

## Trustworthiness

Van Manan is one of the thought leaders in interpretive phenomenology and has described a 6-step approach as shown in Table 2.^13^ Some authors say that they used this approach to guide data analysis; however, the steps guide the whole approach to the methods. The traditional criteria for trustworthiness in qualitative studies can be applied to phenomenology, as shown in Table 3.^14^

**TABLE 2:** Six steps to doing interpretive phenomenology.^13^

Steps	Explanation
1. Turning to the nature of lived experience	Ensure that your study asks a research question about people’s lived experiences. Identify clearly the phenomenon you are interested in.
2. Investigating experience as we live it	Engage sufficiently with the lived experiences of participants; explore the complexity of these experiences in depth, usually via interviews.
3. Reflecting on the essential themes which characterise the phenomenon	Analyse the data to identify and reflect on the essential themes that capture the meaning of the lived experience.
4. Describing the phenomenon in the art of writing and rewriting	Describe the themes in a narrative. The researcher may write and re-write their interpretation as they revisit the data in an iterative process.
5. Maintaining a strong and orientated relation to the phenomenon	The narrative should be aligned with the research question, the data and the researcher’s interpretation.
6. Balancing the parts and the whole	The researcher must balance attention to the individual stories and experiences with attention to the overarching meaning of the whole experience.

**TABLE 3:** Qualitative criteria for trustworthiness applied to phenomenology.^14^

Criteria	Description
Credibility	How coherent is the narrative with the underlying data and the phenomenon of interest? The key question is how ‘valid’ the findings are. Credibility may be improved by appropriate methods, sufficient engagement with the phenomenon, respondent validation and peer review of the process.
Transferability	How transferable the findings are to another setting will depend on how fully the researcher has described the setting or context and the participants.
Dependability	The key question is how ‘reliable’ the findings are. Would you report the same themes if the study was repeated in the same context and period? A very clear and detailed description of the methods would enable someone else to follow you.
Confirmability	Although the researcher does not attempt to bracket themselves from the process, they should demonstrate awareness of their own experiences, assumptions and presuppositions in the reporting.

De Witt has also attempted to provide a framework for the critical appraisal of rigour in interpretive phenomenology, as shown in Table 4.^15^ While these elements are embedded in the phenomenological approach, they do not all seem easy or practical to implement. Resonance and actualisation cannot be judged without engaging current or future readers of the work, and even then, it is not clear how these would be determined. Balanced integration appears to expect that the methods and findings will align with the key principles of phenomenology, derived from the original philosophers. Openness and concreteness appear more applicable to judging the rigour of a manuscript or report.

**TABLE 4:** Framework for rigour in interpretive phenomenology.^15^

Element	Description
Balanced integration	Three aspects include the articulation of the philosophical background and its fit with the researcher and research topic; the integration of these concepts in the methods and findings; and a balance between the voice of the participants and the philosophical explanation.
Openness	Being open and explicit about all the methodological decisions taken in designing and implementing the study.
Concreteness	Findings provide concrete examples of the lived experiences that the reader can relate to from their own experiences.
Resonance	The extent to which the findings have an experiential effect on the reader so that they intuitively have insight into the phenomenon.
Actualisation	The future realisation of the resonance of the study findings.

## Conclusion

The field of phenomenology has different traditions, and it may be difficult to completely hold fast to one approach. The field is defined by a focus on exploring people’s lived experiences, rather than their ideas, beliefs, or perspectives. Primary care researchers may struggle with the underlying philosophical debates while seeking practical guidance on how to conduct rigorous phenomenology. This article provides an overview of the research paradigm while outlining the practical steps needed to conduct primary care and family medicine research.
